# Knowledge, Attitudes, and Factors Associated with Organ Donation Willingness among Adults in Lebanon

**DOI:** 10.1177/15269248261471006

**Published:** 2026-07-23

**Authors:** Lynn Cheaito, Rouba Boukaily, Khaled Mouchref, Sarah Al Banna, Georges Hatem, Fatima Atoui, Dana Cheaito, Sanaa Awada

**Affiliations:** 1 Clinical and Epidemiological Research Laboratory, Faculty of Pharmacy, 63573 Lebanese University, Hadat, Lebanon; 2 Center of research for information technology and communications, Faculty of Engineers, Arts, Sciences and Technology University, Beirut, Lebanon; 3 Laboratoire de Technologie et Instrumentation pour la santé, Lebanese University, Tripoli, Lebanon; 4 Environmental Health Department, National Institute of Health Dr Ricardo Jorge, Porto, Portugal; 5 EPIUnit - Instituto de Saúde Pública, Universidade do Porto, Porto, Portugal; 6 Laboratório para a Investigação Integrativa e Translacional em Saúde Pública (ITR), Porto, Portugal

## Abstract

**Introduction:**

Organ donation (OD) is a life-saving intervention, but its success depends on public willingness to donate.

**Aim:**

This study aimed to assess the knowledge, attitudes, and composite willingness of the Lebanese general public toward OD, and to explore conditions influencing donation decisions.

**Design:**

A cross-sectional study was conducted in Lebanon (December 2024-June 2025) using an online questionnaire distributed via snowball sampling. Knowledge, attitudes, and willingness toward OD were assessed using three carefully constructed and validated scales.

**Results:**

The study included 830 participants, of whom 503 (60.6%) were females. Nearly all participants (97.6%) had heard of OD, but only 20.2% demonstrated good knowledge; 66.7% had a positive attitude (PA); and 43.9% demonstrated favorable composite willingness toward OD, whereas only 34.9% reported current willingness to register as organ or tissue donors. Higher odds of favorable composite willingness were observed among postgraduates (Adjusted Odds Ratio (aOR)=1.75, 95% confidence interval (CI)=[1.12-2.74]), females (aOR=1.66, 95%CI= [1.21-2.26]), workers in the medical/health field (aOR=2.14, 95%CI=[1.55-2.95]), and those employed in the private sector (aOR=2.02, 95%CI=[1.24-3.28]). Common barriers included family and social pressures, fear of discussing death, lack of knowledge, and religious concerns.

**Conclusions:**

Among this predominantly young, educated, and digitally connected Lebanese population, PAs toward OD were common, whereas knowledge and current willingness to register as an organ donor remained limited. Participants reported greater willingness under several hypothetical facilitating conditions, suggesting that informational, social, and structural factors may influence OD decisions. These findings may help inform future strategies to promote OD in Lebanon.

## Introduction

Human organs undergo various changes, some reversible through the body's natural regulatory mechanisms, and others irreversible, potentially leading to organ failure.^1^ Organ donation (OD) is one of the greatest medical advancements, being a life-saving solution for patients suffering from irreversible organ failure.^2^ By definition, OD is the process of giving one's organs or tissues to someone in need of a transplant.^3^ Donations may occur either from a Living Donor (LD), who provides cells, tissues, or organs, or from a Deceased Donor (DD), declared dead according to established medical criteria, from whom organs are recovered for transplantation.^4^ Thus, one donor can potentially save up to eight lives and improve the quality of life of many others.^5^ Despite medical advancements, the demand for transplantable organs far exceeds supply, leading to a persistent global shortage.^2^ The World Health Organization estimates that only 10% of the worldwide demand is currently met. In 2023, a total of 172,397 organ transplants were performed worldwide.^6^ Consent systems play a major role in shaping donation rates. In explicit consent systems, used in countries such as the United States, Canada, Germany, Denmark, and Japan, individuals must actively register their willingness to donate, typically through a donor card or national database. In contrast, presumed consent systems, as adopted in Spain, France, Italy, Portugal, and Belgium, consider all citizens potential donors unless they have formally opted out.^7,8^ However, donation rates are influenced by multiple factors beyond consent legislation, including healthcare infrastructure, transplant coordination, public awareness, and cultural norms. In 2023, Spain reported the highest rate of DDs (49.38 per million population (PMP)), while Turkey had the highest rate of LDs (55.37 PMP).^9^

In Lebanon, OD is regulated by Law Number 109/1983, which requires family consent even if the deceased had previously expressed a willingness to donate.^10^ The National Organization for Organ and Tissue Donation and Transplantation in Lebanon (NOD-Lb), established in 1999 under the Ministry of Health, is the only official body authorized to oversee all organ, tissue, and cell donation and transplantation activities in the country.^11^ NOD-Lb ensures that donations follow strict medical criteria, are transparent, and are fair. However, Lebanon still records fewer than two donors PMP annually, among the lowest rates worldwide.^10^ According to NOD-LB, although 3200 individuals are registered as organ donors, around 700 patients remain on the transplant waiting list.^11,12^ Experts have estimated that the country's deceased donor rate could reach approximately 14 PMP under optimal conditions, potentially increasing the number of life-saving transplantations annually. However, public awareness and consent toward OD remain suboptimal, with studies highlighting the need for educational and policy initiatives to enhance willingness and family authorization for donation in Lebanon.^10,13,14^

Knowledge and attitudes have consistently been reported as factors associated with OD. In Saudi Arabia, organ donor registration has been associated with cultural and family influences, religious beliefs, body integrity concerns, and medical mistrust.^15^ In Tunisia, while OD is widely recognized as important, research has reported that low awareness, cultural barriers, and mistrust in the medical system are associated with lower registration rates.^16^ Lebanon faces similar challenges. A national cross-sectional study found that supportive attitudes were associated with greater knowledge, and that willingness to donate increased under hypothetical scenarios in which religion and law supported the practice. Demographic factors such as age, sex, place of residence, and income were also associated with attitudes.^10^ Understanding the Lebanese public's knowledge, attitudes, and willingness toward OD is therefore essential to address the national shortage and strengthen the donation program. Specifically, limited updated evidence is available regarding the specific barriers and facilitating conditions that may influence donation decisions among Lebanese adults. A better understanding of these factors could help inform targeted awareness campaigns and public health strategies to improve OD acceptance and registration. Therefore, this study aimed to assess knowledge, attitudes, and composite willingness toward OD (including current willingness and willingness under facilitating conditions) among Lebanese adults.

## Design/Methods

### Design

This study employed a cross-sectional observational design and was conducted in Lebanon between December 2024 and June 2025. Ethical approval for this study was obtained from the Institutional Review Board of the Faculty of Pharmacy, Lebanese University (reference:20/S/2024). All procedures were conducted in accordance with the ethical principles outlined in the Declaration of Helsinki. Before participation, all respondents were informed about the study's purpose, and they provided informed consent. They were assured that their participation was voluntary, anonymous, and confidential.

### Population and Setting

The target population consisted of Lebanese adults residing in Lebanon. National demographic indicators indicate that Lebanon has a predominantly young adult population, with approximately 55-60% of the population under 40 years of age. Females comprise slightly more than half of the adult population, and the country has a diverse religious composition, with Muslims and Christians representing the majority, as well as smaller Druze and other communities. Educational attainment in Lebanon is relatively high, with many adults completing secondary or higher education, and a significant proportion engaged in higher education or service-sector occupations. The study's accessible population consisted of Lebanese adults aged 18 or older with active internet access and reachable via digital communication channels during the data collection period. This group represents a technologically connected subset of the broader adult population, more likely to be younger, literate, and regularly engaged with online platforms. Consequently, the study findings should be interpreted in the context of this accessible population. The study was carried out in the community across various geographic areas of Lebanon. Data collection was conducted online via an electronic survey, allowing participants to complete the questionnaire at their convenience rather than in a healthcare or institutional setting.

### Sampling

A snowball sampling technique was used to recruit participants through an online survey disseminated across different Lebanese regions using social media platforms and electronic messaging applications. The inclusion criteria were Lebanese nationality, age ≥18 years, and provision of informed consent. Individuals unable to complete the questionnaire due to cognitive limitations were excluded. The sample size (n) was calculated using Cochran's Sample Size Formula, based on the following parameters: Z, the critical value for a 95% confidence interval (CI); p, the expected prevalence of willingness to donate organs (21.6%)^10^; and e, the margin of error (3%): n = Z^2^×p × (1−p)/e^2^. Based on these parameters, the minimum required sample size was approximately 791 respondents. To enhance the study's reliability and account for potential non-response, 830 participants were ultimately included in the analysis. Participants accessed the online survey via a link distributed through social media platforms and personal networks. After reading the introduction and providing informed consent, participants completed the questionnaire independently. No further interaction or intervention was required from the research team.

### Data Collection

Data were collected using a self-designed questionnaire, developed based on previous research.^17,18^ The questionnaire was translated, culturally adapted, and reviewed for linguistic appropriateness before administration. It was designed to assess the knowledge, attitudes, composite willingness, and factors associated with OD decisions among Lebanese adults, including potential barriers and motivators that may influence donation decisions. The questionnaire was pilot-tested on 5% of the participants to assess its understandability and reliability. Individuals involved in the pilot study were excluded from the main study. The questionnaire was structured into six sections. The introduction provided a brief overview of the study objectives, emphasized the voluntary nature of participation, and assured confidentiality, anonymity, and informed consent. The sociodemographic characteristics section included variables such as sex, age, employment status, field of work, perceived economic status, education level, marital status, religious affiliation, and the importance of religious beliefs. The personal background related to OD assessed familiarity with OD using “Yes,” “No,” or “Don’t know” responses.

The knowledge about the OD section comprised 20 items with “True,” “False,” or “Don’t know” options; correct answers were scored 1, and incorrect or “Don’t know” responses were scored 0, resulting in total scores ranging from 0 to 20. Participants scoring <60% (0-11) were classified as having low knowledge (LK), whereas those scoring ≥60% (12-20) were classified as having high knowledge (HK). The attitudes toward the OD section included 13 statements rated on a 5-point Likert scale (Strongly Disagree to Strongly Agree); Agree/Strongly Agree responses were scored 1, and Neutral/Disagree/Strongly Disagree responses were scored 0, yielding a total score of 0-13. Scores <60% (0-7) indicated a negative attitude (NA), while scores ≥60% (8-13) indicated a positive attitude (PA). A composite OD willingness score was calculated by summing six items. The first item assessed participants’ current willingness to register as an organ/tissue donor, whereas the remaining five items assessed willingness under specific facilitating conditions (eg, awareness campaigns, guidance from a trustworthy organization, clarification of religious perspectives, supportive legislation, and family approval). Accordingly, the score reflects both current willingness and willingness under facilitating conditions rather than unconditional willingness alone. Participants were classified as having high (score 4-6) or low (score 0-3) composite willingness toward OD. Because this composite measure incorporates both current willingness and willingness under hypothetical facilitating conditions, it should be interpreted as reflecting overall donation willingness under varying scenarios rather than an individual's unconditional intention to donate. The internal consistency of each domain was assessed using Cronbach's alpha, with good reliability found for knowledge (α=0.83), attitude (α=0.83), and composite willingness (α=0.90).

### Data Analysis

Data collected via Google Forms were exported to Excel and analyzed using the Statistical Package for the Social Sciences (SPSS) Version 25. Descriptive statistics were computed for all variables, with categorical variables presented as frequencies and percentages, and continuous scores as means ± standard deviations. Bivariate analysis was performed using Chi-square tests to examine associations between knowledge, attitude, and composite willingness and other categorical variables; Fisher's exact test was applied when expected cell counts were low. Variables with *P*-values<0.200 in the bivariate analysis of composite willingness were included in univariate and multivariate logistic regression models to examine the associations between participants’ general characteristics and composite willingness to donate. Adjusted odds ratios (aORs) with 95% CIs were reported. Statistical significance was set at *P* < 0.05.

## Results

### Sociodemographic Characteristics

A total of 830 participants were enrolled, comprising 503 females (60.6%) and 327 males (39.4%) (Table 1). The majority (57.2%) were aged 18-30 years, employed in the private sector (35.3%), and working outside the medical or health sciences field (63.9%). Most participants (82.8%) perceived their economic status as low to middle income, while 143 (17.2%) considered it high. Educational attainment varied: 375 (45.2%) held a graduate degree, and 203 (24.5%) were undergraduates. Regarding marital status, most participants (56.3%) were single. In terms of religion, 372 (44.8%) identified as Muslim, and 438 (52.8%) considered religion to be very important, whereas 57 (6.9%) considered it to be not very important.

**Table 1.** Sociodemographic Characteristics of the Study Participants.

Variables		Frequency (N = 830)	Percentage (%)
Sex	Male	327	39.4
	Female	503	60.6
Age (years)	18–30	475	57.2
	31–40	158	19.0
	41–50	122	14.7
	>50	75	9.0
Marital status	Single	467	56.3
	Married	315	38.0
	Divorced	35	4.2
	Widowed	13	1.6
Level of education	Undergraduate	203	24.5
	Graduate	375	45.2
	Postgraduate	243	29.3
	Cannot read or write	9	1.1
Field of work	Neither medical nor health sciences	530	63.9
	Medical or health sciences	300	36.1
Perceived economic status	Low-middle	687	82.8
	High	143	17.2
Employment status	Not employed	115	13.9
	Employed public sector	108	13.0
	Employed private sector	293	35.3
	Self-employed	110	13.3
	Student	177	21.3
	Unable to work	4	0.5
	Other	23	2.8
Religious background	Not mentioned	99	11.9
	Muslim	372	44.8
	Christian	189	22.8
	Druze	170	20.5
Importance of religious beliefs	Neutral	74	8.9
	Not at all important	51	6.1
	Not very important	57	6.9
	Somewhat important	210	25.3
	Very important	438	52.8

### Participants’ Knowledge, Attitude, and Willingness to Donate Organs

Among the 830 participants, most were familiar with OD (97.6%) and transplantation (95.4%). Overall, 79.8% demonstrated LK and only 20.2% showed HK, with a mean score of 7.94 ± 4.42. Table S1 (Supplemental material) shows the participants’ responses to the knowledge statements. Misconceptions were common: 62.5% believed there is an upper age limit of 60 years for donation, 77.6% were unaware of medical conditions affecting eligibility, and 66.6% knew that most religions support OD. Awareness of Lebanon-specific information was also limited: 56.7% were unaware of the country's low donation rate, 73.6% were unaware of the establishment of NOD-Lb, and only 27.3% knew the year of the first kidney transplant. Figure S1 illustrates the participants’ attitudes toward OD. Most participants demonstrated a PA toward OD (66.7%), with a mean attitude score of 8.00 ± 3.21. The majority considered OD ethically acceptable (78.9%) and expressed willingness to donate to family members (73.6%) or after death (57.6%). Regarding factors influencing donation decisions, 74.1% reported that family beliefs did not affect their decision, 60.6% stated that religion did not influence their views on OD, and 23.3% believed the body should remain intact post-mortem. Additionally, 73.6% believed that strengthening cultural values could positively affect attitudes toward OD. Participants’ composite willingness toward OD is presented in Table S2. Overall, 43.9% expressed a favorable composite willingness toward OD, whereas 56.1% demonstrated unfavorable composite willingness. The mean composite willingness score was 2.80 ± 2.41. Only 34.9% reported being currently willing to register as organ or tissue donors. Potential motivators included increased awareness through educational campaigns (51.1%), encouragement by a trustworthy organization (46.5%), and supportive laws or religious guidance (53.1%). Nevertheless, 54.2% reported they would remain unwilling even if their families had no objection. Regarding unwillingness to donate, 66.6% of participants reported one reason, 14.1% two reasons, and 19.3% three or more reasons. The most frequently cited barriers included the absence of financial benefit, family and social pressures, fear of discussing death, fear of not receiving appropriate medical care, lack of knowledge, lack of trust in the allocation process, and religious beliefs.

Table 2 shows the associations between participants’ general characteristics and attitudes toward OD. PAs were more common among participants with HK than among those with PK (85.7% vs 61.9%, *P* < 0.001). Participants working in the medical or health sciences also exhibited a higher proportion of PA than those working in non-medical fields (76.7% vs 61.1%, *P* < 0.001). The proportion of participants with PA was higher among those with higher educational attainment, ranging from 55.7% among undergraduates to 74.9% among postgraduates (*P* < 0.001). Female participants were more likely than male participants to report PA (70.0% vs 61.8%, *P* = 0.014). Differences were also observed by employment status (*P* = 0.028), with private sector employees having the highest proportion of PA (72.0%), whereas public sector employees had the lowest (58.3%). No significant associations were found with age (*P* = 0.309), perceived economic status (*P* = 0.501), marital status (*P* = 0.063), religious background (*P* = 0.591), or the perceived importance of religious beliefs (*P* = 0.875). Table 2 also presents the associations between participants’ characteristics and knowledge about OD. HK was significantly more frequent among participants working in the medical or health sciences field than among those working outside this field (34.0% vs 12.5%, *P* < 0.001). It was more prevalent among self-employed participants (37.3%) and public sector employees (27.8%) than among unemployed participants (17.4%), private sector employees (17.1%), and students (12.4%) (*P* < 0.001). Participants reporting a high perceived economic status were more likely to have HK about OD than those reporting a low-to-middle economic status (49.0% vs 14.3%, *P* < 0.001). The prevalence of HK increased with educational attainment, ranging from 8.9% among undergraduates to 30.5% among postgraduates (*P* < 0.001). HK was significantly more frequent among Christian participants (28.6%) than among participants from other religious backgrounds (*P* = 0.008) and among those reporting that religion was not at all important (45.1%) or not very important (31.6%) compared with those reporting religion as somewhat or very important (*P* < 0.001). Finally, participants with PA toward OD demonstrated a significantly higher prevalence of HK than those with NA (26.0% vs 8.7%, *P* < 0.001). No significant associations were observed with age (*P* = 0.579), sex (*P* = 0.973), or marital status (*P* = 0.082).

**Table 2.** Association Between Participants’ Sociodemographic Characteristics, Attitudes Toward, and Knowledge About Organ Donation.

Variables		Negative Attitude	Positive Attitude	*P*-value
**Age**	18–30 years	155 (32.6)	320 (67.4)	0.309
	31–40 years	53 (33.5)	105 (66.5)	
	41–50 years	48 (39.3)	74 (60.7)	
	>50 years	20 (26.7)	55 (73.3)	
**Sex**	Male	125 (38.2)	202 (61.8)	**0**.**014**
	Female	151 (30.0)	352 (70.0)	
**Employment status**	Unemployed	45 (39.1)	70 (60.9)	**0**.**028**
	Public sector employee	45 (41.7)	63 (58.3)	
	Private sector employee	82 (28.0)	211 (72.0)	
	Self-employed	39 (35.5)	71 (64.5)	
	Student	61 (34.5)	116 (65.5)	
	Unable to work	1 (25.0)	3 (75.0)	
**Field of work**	Non-medical	206 (38.9)	324 (61.1)	**<0**.**001**
	Medical field	70 (23.3)	230 (76.7)	
**Perceived economic status**	Low-middle	225 (32.8)	462 (67.2)	0.501
	High	51 (35.7)	92 (64.3)	
**Level of education**	Undergraduate	90 (44.3)	113 (55.7)	**<0**.**001**
	Graduate	119 (31.7)	256 (68.3)	
	Postgraduate	61 (25.1)	182 (74.9)	
	Cannot read or write	6 (66.7)	3 (33.3)	
**Marital status**	Single	151 (32.3)	316 (67.7)	0.063
	Married	101 (32.1)	214 (67.9)	
	Divorced	16 (45.7)	19 (54.3)	
	Widowed	8 (61.5)	5 (38.5)	
**Religion**	Not mentioned	29 (29.3)	70 (70.7)	0.591
	Muslim	126 (33.9)	246 (66.1)	
	Christian	59 (31.2)	130 (68.8)	
	Druze	62 (36.5)	108 (63.5)	
**The importance of religious beliefs**	Neutral	27 (36.5)	47 (63.5)	0.875
	Not at all important	19 (37.3)	32 (62.7)	
	Not very important	19 (33.3)	38 (66.7)	
	Somewhat important	65 (31.0)	145 (69.0)	
	Very important	146 (33.3)	292 (66.7)	
**Knowledge**	Poor	252 (38.1)	410 (61.9)	**<0**.**001**
	Good	24 (14.3)	144 (85.7)	

Variables		Low Knowledge	High Knowledge	*P*-value
**Age**	18–30 years	384 (80.8)	91 (19.2)	0.579
	31–40 years	20 (75.9)	38 (24.1)	
	41–50 years	99 (81.1)	23 (18.9)	
	>50 years	59 (78.7)	16 (21.3)	
**Sex**	Male	261 (79.8)	66 (20.2)	0.973
	Female	401 (79.7)	102(20.3)	
**Employment status**	Unemployed	95 (82.6)	20 (17.4)	**<0**.**001**
	Public sector employee	78 (72.2)	30 (27.8)	
	Private sector employee	243 (82.9)	50 (17.1)	
	Self-employed	69 (62.7)	41 (37.3)	
	Student	115 (87.6)	22 (12.4)	
	Unable to work	4 (100.0)	–	
**Field of work**	Non-medical	464 (87.5)	66 (12.5)	**<0**.**001**
	Medical field	198 (66.0)	102 (34.0)	
**Perceived economic status**	Low-middle	589 (85.7)	98 (14.3)	**<0**.**001**
	High	73 (51.0)	70 (49.0)	
**Level of education**	Undergraduate	185 (91.1)	18 (8.9)	**<0**.**001**
	Graduate	300 (80.0)	75 (20.0)	
	Postgraduate	169 (69.5)	74 (30.5)	
	Cannot read or write	8 (88.9)	1 (11.1)	
**Marital status**	Single	379 (81.2)	88 (18.8)	0.082
	Married	251 (79.7)	64 (20.3)	
	Divorced	22 (62.9)	13 (37.1)	
	Widowed	10 (76.9)	3 (23.1)	
**Religion**	Not mentioned	85 (85.9)	14 (14.1)	**0**.**008**
	Muslim	301 (80.9)	71 (19.1)	
	Christian	135 (71.4)	54 (28.6)	
	Druze	141 (82.9)	29 (17.1)	
**The importance of religious beliefs**	Neutral	61 (82.4)	13 (17.6)	**<0**.**001**
	Not at all important	28 (54.9)	23 (45.1)	
	Not very important	39 (68.4)	18 (31.6)	
	Somewhat important	176 (83.8)	34 (16.2)	
	Very important	358 (81.7)	80 (18.3)	
**Attitude**	Negative	252 (91.3)	24 (8.7)	**<0**.**001**
	Positive	410 (74.0)	144 (26.0)	

*P*<0.05 are presented in bold.

Table 3 presents the associations between participants’ general characteristics and composite willingness toward OD. Participants with PA were substantially more likely to have high composite willingness than those with NA (59.2% vs 13.0%, *P* < 0.001). Similarly, participants with HK were more likely to have high composite willingness than those with LK (67.3% vs 37.9%, *P* < 0.001). High composite willingness was also more common among participants working in the medical or health sciences than among those working in non-medical fields (58.0% vs 35.8%, *P* < 0.001). Participants with postgraduate education exhibited the highest proportion of high composite willingness (56.8%), whereas undergraduate participants had the lowest (32.5%) (P < 0.001). Composite willingness also differed by employment status (*P* = 0.003), with private sector employees showing the highest proportion of high composite willingness (50.5%) and unemployed participants the lowest (31.3%). Female participants were more likely than male participants to have high composite willingness (47.7% vs 37.9%, *P* = 0.005). Participants who considered religion not at all important (62.7%) or not very important (52.6%) were more likely to have high composite willingness than those who considered religion somewhat (41.4%) or very important (41.1%) (*P* = 0.023). No significant associations were observed with age (*P* = 0.970), perceived economic status (*P* = 0.327), marital status (*P* = 0.494), or religious background (*P* = 0.278).

**Table 3.** Association Between Participants’ Sociodemographic Characteristics and Willingness Toward Organ Donation.

Variables		Low Willingness	High Willingness	*P*-value
**Age**	18–30 years	264 (55.6)	211 (44.4)	0.970
	31–40 years	91 (57.6)	67 (42.4)	
	41–50 years	68 (55.7)	54 (44.3)	
	>50 years	43 (57.3)	32 (42.7)	
**Sex**	Male	203 (62.1)	124 (37.9)	**0**.**005**
	Female	263 (52.3)	240 (47.7)	
**Employment status**	Unemployed	79 (68.7)	36 (31.3)	**0**.**003**
	Public sector employee	61 (56.5)	47 (43.5)	
	Private sector employee	145 (49.5)	148 (50.5)	
	Self-employed	59 (53.6)	51 (46.4)	
	Student	111 (62.7)	66 (37.3)	
	Unable to work	2 (50.0)	2 (50.0)	
**Field of work**	Non-medical	340 (64.2)	190 (35.8)	**<0**.**001**
	Medical field	126 (42.0)	174 (58.0)	
**Perceived economic status**	Low-middle	391 (56.9)	296 (43.1)	0.327
	High	75 (52.4)	68 (47.6)	
**Level of education**	Undergraduate	137 (67.5)	66 (32.5)	**<0**.**001**
	Graduate	218 (58.1)	157 (41.9)	
	Postgraduate	105 (43.2)	138 (56.8)	
	Cannot read or write	6 (66.7)	3 (33.3)	
**Marital status**	Single	262 (56.1)	205 (43.9)	0.494
	Married	174 (55.2)	141 (44.8)	
	Divorced	20 (57.1)	15 (42.9)	
	Widowed	10 (76.9)	3 (23.1)	
**Religion**	Not mentioned	54 (54.5)	45 (45.5)	0.278
	Muslim	213 (57.3)	141 (44.8)	
	Christian	96 (50.8)	15 (42.9)	
	Druze	103 (60.6)	67 (39.4)	
**The importance of religious beliefs**	Neutral	39 (52.7)	35 (47.3)	**0**.**023**
	Not at all important	19 (37.3)	32 (62.7)	
	Not very important	27 (47.4)	30 (52.6)	
	Somewhat important	123 (58.6)	87 (41.4)	
	Very important	258 (58.9)	180 (41.1)	
**Knowledge**	Poor	411 (62.1)	251 (37.9)	**<0**.**001**
	Good	55 (32.7)	113 (67.3)	
**Attitude**	Negative	240 (87.0)	36 (13.0)	**<0**.**001**
	Positive	226 (40.8)	328 (59.2)	

*P*<0.05 are presented in bold.

In the multivariate logistic regression analysis (Table 4), several factors were significantly associated with higher composite willingness toward OD. Participants with postgraduate education had 1.75-fold higher odds of having high composite willingness compared with undergraduate participants (aOR=1.75, 95% CI = [1.12-2.74], *P* = 0.015). Participants working in the medical and health sciences field had 2.14-fold higher odds of having high composite willingness compared to those employed in other sectors (aOR=2.14, 95% CI = [1.55-2.95], *P* < 0.001). Similarly, participants employed in the private sector had 2.02-fold higher odds of having high composite willingness than unemployed participants (aOR=2.02, 95% CI = [1.24-3.28], *P* = 0.005). Additionally, female participants had 1.66-fold higher odds of having high composite willingness toward OD than male participants (aOR=1.66, 95% CI = [1.21-2.26], *P* = 0.002).

**Table 4.** Predictors of Higher Composite Willingness Toward Organ Donation Among Study Participants.

		Crude Model			Adjusted Model		
Variables		OR	95% CI	*P*-value	OR	95% CI	*P*-value
**Sex**	Male	Reference			Reference		
	Female	1.49	1.13–1.96	**0**.**006**	1.66	1.21–2.26	**0**.**002**
**Employment status**	Unemployed	Reference			Reference		
	Public sector employee	1.69	0.98–2.92	0.060	1.69	0.94–3.04	0.077
	Private sector employee	2.24	1.42–3.53	**0**.**001**	2.02	1.24–3.28	**0**.**005**
	Self-employed	1.90	1.10–3.27	**0**.**021**	1.76	0.97–3.18	0.062
	Student	1.30	0.79–2.15	0.295	1.34	0.78–2.29	0.291
	Unable to work	2.19	0.30–16.20	0.441	3.29	0.41–26.28	0.261
**Field of work**	Non-medical	Reference			Reference		
	Medical field	2.47	1.85–3.30	**<0**.**001**	2.14	1.55–2.95	**<0**.**001**
**Level of education**	Undergraduate	Reference			Reference		
	Graduate	1.50	1.05–2.14	**0**.**028**	1.20	0.80–1.79	0.381
	Postgraduate	2.73	1.85–4.02	**<0**.**001**	1.75	1.12–2.74	**0**.**015**
	Cannot read or write	1.04	0.25–4.28	0.959	0.94	0.21–4.25	0.930
**The importance of religious beliefs**	Neutral	Reference			Reference		
	Not at all important	1.88	0.91–3.89	0.090	1.64	0.76–3.53	0.210
	Not very important	1.24	0.62–2.47	0.545	1.11	0.54–2.29	0.779
	Somewhat important	0.79	0.46–1.34	0.381	0.59	0.33–1.03	0.064
	Very important	0.78	0.47–1.28	0.318	0.59	0.35–1.00	0.051

Note: OR, odds ratio; CI, confidence interval; *P* < 0.05 are presented in bold.

## Discussion

Understanding public knowledge and attitudes toward OD is important for identifying misconceptions and informing awareness strategies that may support informed decision-making regarding OD. Nearly all participants (97.6%) reported hearing about OD, which is consistent with a similar Lebanese study that found approximately 100% of people had heard about it.^10^ Such high awareness levels suggest that OD is not an unfamiliar concept in Lebanon. However, awareness did not necessarily translate into adequate understanding, as HK about OD was limited to only 20.2% in this study, compared with 34.1% reported among university students in Oman.^18^ This difference should be interpreted cautiously because the Omani study was conducted exclusively among university students, whereas the present study included adults from the general population, making direct comparisons less straightforward.

Knowledge related to religious perspectives on OD showed that 66.6% of participants believed that most religions do not support OD. Although the proportions differ, both this study and a Moroccan study, where 37.5% of participants considered OD prohibited by Islam, suggest that misconceptions regarding religious perspectives may represent an important barrier to OD.^19^ Providing accurate information regarding the position of major religions on OD may therefore help address this misconception. Participants working in the medical or health sciences fields demonstrated significantly higher knowledge levels (*P* < 0.001). Similar patterns have been reported in Oman, where HK was observed among students from the Colleges of Medicine and Health Sciences (46.7%) and Nursing (45.7%),^18^ and in Egypt,^20^ where the mean knowledge score was significantly higher among medical than non-medical students. This finding may reflect differences in educational background and professional exposure to health-related topics. However, because of the cross-sectional design of the present study, the underlying reasons for this association cannot be established.

In terms of attitude, 66.7% of the participants demonstrated a PA toward OD, a higher proportion than that reported among Omani students (29.8%).^18^ Differences in study populations, educational backgrounds, and cultural contexts may therefore have contributed to the observed variation. Despite the relatively high prevalence of PAs, only 34.9% of participants reported being currently willing to register as organ or tissue donors, highlighting a gap between favorable attitudes and current registration intentions. Although the present study did not find sex differences in knowledge, females demonstrated significantly higher attitudes toward OD (*P* = 0.014), a finding consistent with Jordanian data (*P* = 0.004).^21^ While the reasons underlying this association cannot be determined from the present study, sociocultural factors and differences in prosocial attitudes may have contributed to this pattern.

In this study, 34.9% of participants reported being currently willing to register as an organ or tissue donor, which is lower than the rates reported in Tunisia (80.7%)^22^ and slightly lower than in China (47.5%).^23^ Nevertheless, OD systems, legislative frameworks, healthcare infrastructure, and public awareness initiatives may differ substantially across countries. For example, Tunisia operates under a presumed consent system, which, together with public awareness initiatives and other contextual factors, may contribute to higher reported willingness. Similarly, differences in healthcare systems, public trust, and OD policies may partly explain the slightly higher willingness reported in China. Religious importance was statistically associated with a composite willingness toward OD (*P* = 0.023), suggesting that religious beliefs remain an important consideration in donation decisions. Similar findings have been reported in several countries within the region. In Syria, religious beliefs were the primary reason for refusal, despite a 2001 decision by the religious authorities allowing OD, which remains largely unknown to the public.^24^ In Tunisia, over 55.4% of those who refused donation cited religious reasons despite the support of Islamic authorities for OD.^22^ Similarly, in Saudi Arabia, religion significantly influenced decision-making, and participants who were aware of the fatwa permitting OD demonstrated greater acceptance (*P* = 0.001).^25^ These findings suggest that misconceptions regarding religious perspectives, rather than religious doctrine itself, may represent an important barrier to OD in several Eastern Mediterranean countries.

An important finding of this study is that participants’ willingness to donate organs was not static but varied according to specific facilitating conditions. While only 34.9% reported current willingness to register as organ donors, this proportion increased when participants considered scenarios involving supportive legislation and religious endorsement, awareness campaigns and educational initiatives, clarification of religious perspectives, engagement by a trustworthy organization, or family support. These findings indicate that participants’ willingness toward OD differed across hypothetical scenarios, suggesting that informational, social, and structural factors may be relevant considerations in donation decisions. Accordingly, future interventions incorporating public education, engagement with religious leaders, supportive policies, and communication through trusted organizations warrant further evaluation as potential approaches to promote OD.

Concerns about bodily integrity after death remain an important barrier to OD in the Eastern Mediterranean region. In Lebanon, 76.7% of participants were neutral or believed the body should remain intact, and 71.7% feared disfigurement, which may partly reflect misconceptions regarding organ retrieval procedures. In contrast, only 22.3% of participants in India expressed such fears, although this comparison should be interpreted cautiously because cultural, religious, and healthcare contexts differ substantially between the two countries.^26^ These findings highlight the potential influence of cultural, religious, and educational contexts on attitudes toward OD. Moreover, knowledge was significantly associated with composite willingness toward OD (*P* < 0.001). Furthermore, 51.1% reported that they would consider OD if additional awareness campaigns and educational programs were implemented. Similar trends have been observed across the region, where inadequate knowledge was the most cited barrier in Oman (58.8%),^18^ Jordan (79.5%),^21^ and Saudi Arabia.^25^ A Lebanese study also reported higher willingness among participants with greater knowledge and awareness. Collectively, these findings suggest that knowledge may represent an important factor associated with willingness toward OD, highlighting the urgent need for targeted educational programs to enhance acceptance rates both in Lebanon and across the Arab world.^10,27–29^ A strong association was found between attitude and composite willingness to donate organs (*P* < 0.001). This aligns with previous research conducted in Lebanon, where PA was significantly associated with greater willingness to donate after adjusting for potential confounders (*P* < 0.001).^10^ Similarly, a study among university students in the United Arab Emirates reported that participants with favorable attitudes, including the belief that donating is a noble act, had higher odds of willingness to donate (aOR=4.68).^30^ These findings suggest that PAs are consistently associated with greater willingness toward OD across different populations.

This study has several strengths. It provides updated evidence on knowledge, attitudes, and willingness toward OD among adults in Lebanon, an area where contemporary population-based data remain limited. The large sample size improved the precision of the estimated associations and provided adequate statistical power to examine factors associated with knowledge, attitudes, and composite willingness toward OD. The online survey facilitated rapid, efficient data collection while enabling the recruitment of participants across Lebanon, thereby enhancing the geographic diversity of the sample. However, several limitations should be considered. The cross-sectional design precludes causal inference. The use of an online survey with snowball sampling may have introduced selection bias by preferentially recruiting individuals who were younger, more educated, and digitally connected. Consequently, the findings should be interpreted as reflecting this segment of the Lebanese population rather than the broader Lebanese adult population. The reliance on self-reported data may have introduced social desirability bias, particularly for questions about attitudes and willingness to donate. In addition, the composite willingness toward OD measure combined one item assessing current willingness to register as an organ donor with five items assessing willingness under hypothetical facilitating conditions. Although this approach was intended to capture a broader spectrum of willingness toward OD, it may have overestimated unconditional willingness to donate. Finally, information on participants’ geographic region was not collected, preventing the assessment of potential regional differences, and some educational categories included relatively few participants, reducing the precision of subgroup analyses. Certain participant groups (eg, individuals unable to read or write) were represented by small sample sizes, which may have limited the precision of the corresponding estimates despite the use of appropriate statistical methods for sparse data. Future research should aim to recruit more representative samples of the Lebanese population using probability-based or mixed recruitment strategies. Longitudinal and interventional studies are also warranted to evaluate whether strategies such as public education, engagement with religious leaders, and supportive policy initiatives are associated with improvements in OD knowledge, attitudes, and willingness. Such studies would help validate these findings and better inform national policies and awareness initiatives related to OD.

## Conclusions

While knowledge regarding OD remains limited, PAs were relatively common among Lebanese adults. However, these favorable attitudes were not fully reflected in current willingness to register as organ donors, suggesting that positive perceptions alone were not consistently associated with donor registration intentions. Importantly, participants’ willingness was higher under several hypothetical facilitating conditions, particularly those involving supportive legislation and religious endorsement, awareness campaigns, and clarification of religious perspectives. In addition to the observed associations between knowledge, attitudes, and composite willingness, these findings suggest that informational, social, and structural barriers may play an important role in OD decisions. Future studies should evaluate whether interventions that incorporate public education, engagement with religious leaders, supportive policies, and communication through trusted organizations can increase willingness to donate organs and to register as organ donors.

## Supplemental Material

sj-docx-1-pit-10.1177_15269248261471006 - Supplemental material for Knowledge, Attitudes, and Factors Associated with Organ Donation Willingness among Adults in Lebanon Supplemental material, sj-docx-1-pit-10.1177_15269248261471006 for Knowledge, Attitudes, and Factors Associated with Organ Donation Willingness among Adults in Lebanon by Lynn Cheaito, Rouba Boukaily, Khaled Mouchref, Sarah Al Banna, Georges Hatem, Fatima Atoui, Dana Cheaito and Sanaa Awada in Progress in Transplantation
