# Supplementary material for: Knowledge, Attitudes, and Factors Associated with Organ Donation Willingness among Adults in Lebanon
Source: Prog Transplant. 2026 Jul 23;36(3):122–31. doi: 10.1177/15269248261471006 (PMC13433881; doi:10.1177/15269248261471006)
Supplement: Supplementary file 1 [file sj-docx-1-pit-10.1177_15269248261471006.docx]

**Knowledge, attitudes, and willingness toward organ donation among the general public in Lebanon: a cross-sectional study**

**Figure S1**. Participants' attitudes toward organ donation

|  | Neutral/Disagree/Strongly Disagree | Strongly Agree/Agree |
| --- | --- | --- |
| Do you agree tha OD is ethically right | 21.1 | 78.9 |
| Do you agree to donate organs after your death | 42.4 | 57.6 |
| Do you agree to donate organs to your family member's | 26.4 | 73.6 |
| Is your decision about OD influenced by your family beliefs | 74.1 | 25.9 |
| Do you agree that donating organs adds meaning to one’s life | 23.6 | 76.4 |
| Does your religion support OD or transplantation | 60.6 | 39.4 |
| Do you believe your body should remain intact after death | 76.7 | 23.3 |
| Are you afraid that donating organs will disfugure your body | 71.7 | 28.3 |
| Do you think more cultural awareness can change people's attitudes | 26.4 | 73.6 |
| Do yout hink OD is atreatment option | 18.6 | 81.4 |
| Do you think OD can save someone’s life | 12.3 | 87.7 |
| Do you know you can donate one of your kidneys or part of your liver while alive | 14.3 | 85.7 |
| Do you knowt hat donating an organ or tissue is safe | 31.7 | 68.3 |

Table S1. Knowledge Assessment among the Lebanese General Public

| **Statement** | **Answers** | **Frequency (%)** |
| --- | --- | --- |
| **Item 1:** To become an organ donor, you must agree to donate all of your organs | True/don’t know | 153 (18.4) |
|  | False | 677 (81.6) |
| **Item 2:** There is an upper age limit of 60 for organ donation | True/don’t know | 519 (62.5) |
|  | False | 311 (37.5) |
| **Item 3:** There is no age limit for donation or to sign up to the organ donor register | True | 497 (59.9) |
|  | False/don’t know | 333 (40.1) |
| **Item 4:** Organ donation prevents the family from viewing the deceased or having an open-casket funeral | True/don’t know | 434 (52.3) |
|  | False | 396 (47.7) |
| **Item 5:** Organs and tissues donated for transplantation cannot be used for medical research without appropriate consent | True | 561 (67.6) |
|  | False/don’t know | 269 (32.4) |
| **Item 6**: Carrying an organ donor card alone is sufficient to ensure that your wish to donate will always be carried out | True | 445 (53.6) |
|  | False/don’t know | 385 (46.4) |
| **Item 7:** People of all ethnic and religious backgrounds may need an organ transplant | True | 133 (16.0) |
|  | False/don’t know | 697 (84.0) |
| **Item 8:** Family consent is often required before organs can be donated after death in Lebanon | True/don’t know | 533 (64.2) |
|  | False | 297 (35.8) |
| **Item 9:** If donated organs are not suitable for transplantation, they are automatically used for medical research | True/don’t know | 645 (77.7) |
|  | False | 185 (22.3) |
| **Item 10:** You can be a living organ donor. While alive, you can donate one kidney, one lung, part of the liver, islets of the pancreas, or bone marrow. | True | 614 (74.0) |
|  | False/don’t know | 216 (26.0) |
| **Item 11:** Brain-dead donors can donate only the following organs: Kidneys, liver, heart, lungs, and pancreas | True/don’t know | 687 (82.8) |
|  | False | 143 (17.2) |
| **Item 12:** Brain donation is only used to advance scientific research, instead of helping another person stay alive through a transplant | True | 272 (32.8) |
|  | False/don’t know | 558 (67.2) |
| **Item 13:** Spreading cancer, systemic infection, transmissible disease (e.g., Hepatitis, AIDS), or heart disease are the only conditions that may render a person ineligible to donate organs | True/don’t know | 644 (77.6) |
|  | False | 188 (22.4) |
| **Item 14:** Living donation has the same process as that of a deceased donor donation | True/don’t know | 493 (59.4) |
|  | False | 337 (40.6) |
| **Item 15:** The organ donor’s family is charged for the donation and should pay all costs related to organ donation | True/don’t know | 390 (47.0) |
|  | False | 440 (53.0) |
| **Item 16:** Most religions support organ and tissue donation as generous acts that benefit people. | True | 553 (66.6) |
|  | False /don’t know | 277 (33.4) |
| **Item 17:** Lebanon has one of the lowest rates of organ donation compared to other countries in the world, where it is less than 2 donors per million people a year | True | 359 (43.3) |
|  | False /don’t know | 471 (56.7) |
| **Item 18:** In Lebanon, the first organ transplant occurred in 1972. It was a kidney transplant undertaken with the help of a living donor at the Barbir hospital | True | 227 (27.3) |
|  | False /don’t know | 603 (72.7) |
| **Item 19:** Signing up to be an organ or tissue donor will also include brain donation for medical and scientific purposes, which is legalized by the Lebanese Law | True | 180 (21.7) |
|  | False /don’t know | 650 (78.3) |
| **Item 20:** NOD-LB was founded in 1999 with a joint decision by the MOH and the Lebanese Order of Physicians (LOP) | True | 219 (26.4) |
|  | False /don’t know | 611 (73.6) |

NOD-LB: National Organization for Organ and Tissue Donation and Transplantation in Lebanon. Results are given in terms of frequency (percentage)

Table S2. Participants' Responses to Willingness Questions

| **Questions** | **Answers** | **Frequency (%)** |
| --- | --- | --- |
| **Item 1:** Are you willing to register as an organ/tissue donor? | No/don’t know | 540 (65.1) |
|  | Yes | 290 (34.9) |
| **Item 2:** Would you consider organ donation if more awareness campaigns and educational programs were done? | No/don’t know | 406 (48.9) |
|  | Yes | 424 (51.1) |
| **Item 3:** Would you consider donating organs more seriously if approached by a trustworthy organization? | No/don’t know | 444 (53.5) |
|  | Yes | 386 (46.5) |
| **Item 4:** Would you consider organ donation if more information were available about your religion's perspective on the matter? | No/don’t know | 425 (51.2) |
|  | Yes | 405 (48.8) |
| **Item 5:** If both laws and religion encourage donation, will you donate? | No/don’t know | 389 (46.9) |
|  | Yes | 441 (53.1) |
| **Item 6**: Would you be willing to register as an organ donor if your family had no objection to the donation of your organs after death? | No/don’t know | 450 (54.2) |
|  | Yes | 380 (45.8) |

Results are given in terms of frequency (percentage)
